# MicroRNA-107 contributes to post-stroke angiogenesis by targeting Dicer-1

**DOI:** 10.1038/srep13316

**Published:** 2015-08-21

**Authors:** Yanan Li, Ling Mao, Yuan Gao, Suraj Baral, Yifan Zhou, Bo Hu

**Affiliations:** 1 Department of Neurology, Union Hospital, Tongji Medical College, Huazhong University of Science and Technology, Wuhan 430022, China

## Abstract

Previous studies have suggested that microRNA-107 (miR-107) regulates cell migration in tumor and promotes Hypoxia Inducible Factor 1α (HIF1α) regulated angiogenesis under hypoxia. We found that miR-107 was strongly expressed in ischemic boundary zone (IBZ) after permanent middle cerebral artery occlusion (pMCAO) in rats and inhibition of miR-107 could reduce capillary density in the IBZ after stroke. Such finding led us to hypothesize that miR-107 might regulate post-stroke angiogenesis and therefore serve as a therapeutic target. We also found that antagomir-107, a synthetic miR-107 inhibitor, decreased the number of capillaries in IBZ and increased overall infarct volume after pMCAO in rats. We demonstrated that miR-107 could directly down-regulate Dicer-1, a gene that encodes an enzyme essential for processing microRNA (miRNA) precursors. This resulted in translational desupression of VEGF (vascular endothelial growth factor) mRNA, thereby increasing expression of endothelial cell-derived VEGF (VEGF165/VEGF164), leading to angiogenesis after stroke. This process might be a protective mechanism for ischemia-induced cerebral injury and miR-107 might be used as a novel tool in stroke treatment.

It is now generally accepted that angiogenesis is critical to post-ischemia response and recovery after stroke^1^. Recently, a number of studies demonstrated that a variety of miRNAs are involved in angiogenesis following ischemic insult^2^,^3^. Studies show that miR-107 was linked to angiogenesis^4^ and was one of the Hypoxia responsive microRNAs (HRMs) induced by HIF1α under hypoxia in tumor^5^,^6^. But whether miR-107 exerts any effect on the angiogenesis after stroke remains less clear.

In this study, we found that miR-107 was significantly up-regulated after pMCAO in rats and inhibition of miR-107 resulted in reduced capillary density in IBZ. Moreover, we showed that both endothelial cells (ECs) and astrocytes secreted miR-107 under hypoxia, but the up-regulated miR-107 acted mainly on ECs to promote tube formation.

Recent studies revealed that VEGF secreted by endothelial cells are functionally different from VEGF secreted by astrocytes^7^. Endothelial cell-derived VEGF maintains endothelial homeostasis^8^ and vascular homeostasis^9^. Endothelial cell-specific deletion of VEGF clearly inhibited human umbilical vein endothelial cells (HUVECs) tube formation^8^. However, it was found that astrocytes-derived VEGF disrupts tight junctions of cortical endothelial cells via claudin-5 (CLN-5) and occludin (OCLN), thereby damaging blood-brain barrier in central nervous system (CNS)^10^ and is essential for pathological, not developmental, retinal angiogenesis^11^. In this study, we observed that it was endothelial cell-derived VEGF165, not astrocytes-derived VEGF165, which was regulated by miR-107 to promote ECs tube formation and migration. Subsequently, dual-luciferase reporter system revealed that Dicer-1 was a direct target of miR-107. Finally, using lentivirus anti-miR-107 and anti-Dicer-1 transfected HUVECs, we demonstrated that miR-107 increased endogenous VEGF165 (VEGF164) via Dicer-1.

## Results

### Increased miR-107 in IBZ promotes angiogenesis in rat after pMCAO

**Figure 1:**
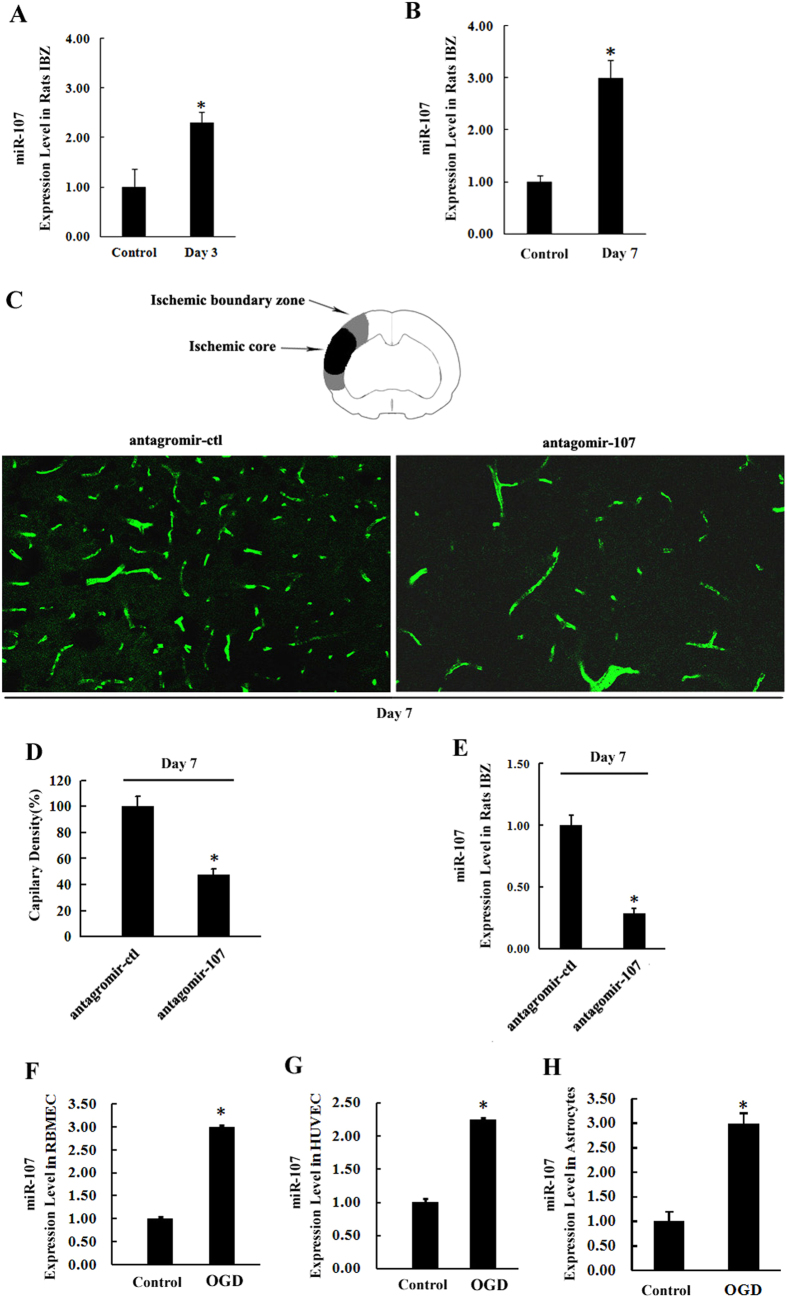
Increased miR-107 in IBZ promotes angiogenesis in rat after pMCAO. (**A**,**B**) qRT-PCR showed the expression of miR-107 was increased in the IBZ of rats subjected to pMCAO on 3rd and 7th day. *P < *0.05*, vs. control group. (**C**) Capillary density was evaluated by FITC tail vein injection, and then the vessel number was quantified by FITC (green). (**D**) Quantification of capillary density. Data are presented as mean ± SD. Scale bar = 10 μm in D (applies to C). *P < *0.05*, vs. antagomir-ctl. (**E**) qRT-PCR showed the expression of miR-107 after lateral cerebral ventricle injection of antagomir-107 on day 7 after pMCAO. *P < *0.05*, vs. antagomir-ctl. (**F**) Exposure to OGD for 12 h increases miR-107 expression in RBMECs demonstrated by using qRT-PCR. (**G**) HUVECs. (**H**) Astrocytes. Data are presented as mean ± SD. *P < *0.05,* vs. control group.

The expression of miR-107 was up-regulated by 2.21 fold and 2.88 fold (Fig. 1A,B, *P* < *0.05*) on day 3 and day 7 respectively in the IBZ of pMCAO rats compared to control as detected by quantitative real-time PCR (qRT-PCR). Quantitative evaluation showed that blockage of miR-107 by lateral ventricular injection of antagomir-107 in pMCAO rats (Fig. 1E, *P* < *0.05*) resulted in reduction of number of capillaries in IBZ by 65.4% as compared with antagomir control group (antagomir-ctl) (Fig. 1C,D, *P* < *0.05*). qRT-PCR showed that miR-107 level was upregulated significantly by 2.99 fold, 2.24 fold and 2.96 fold in rat brain microvascular endothelial cells (RBMECs), HUVECs and astrocytes respectively under Oxygen- Glucose Deprivation (OGD) compared with normoxia after 12 hours. (Fig. 1F–H, *P* < *0.05*).

### miR-107 enhances tubular formation and migration of RBMECs and HUVECs *in vitro*

**Figure 2:**
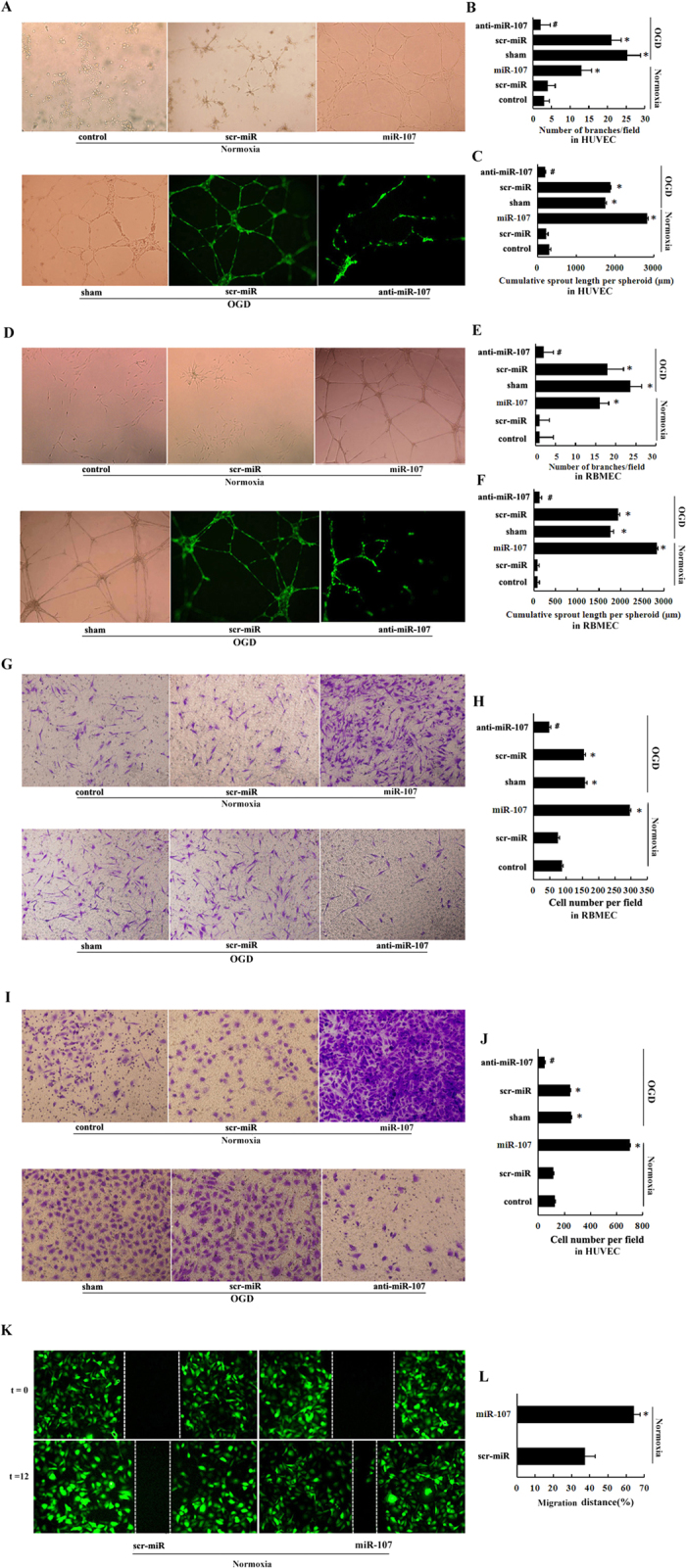
miR-107 enhances tubular formation and migration of RBMECs and HUVECs *in vitro.* (**A**) Top: Representative photomicrographs of tube formation of HUVECs transfected with miR-107 or scr-miR and un-transfected HUVECs (control). Tube formation was measured after 12 hours under normoxia. Bottom: Tube formation capacity of HUVECs transfected with anti-miR-107 or scr-miR and un-transfected HUVECs (sham). The anti-miR-107 effect was investigated under OGD for 12 h. Total magnification, ×100 (**B)** the number of tubule branches per field. (**C**) The cumulative sprout length per field. (**D**–**F**) RBMECs (**G**) Top: RBMECs transfected with miR-107 or scr-miR and un-transfected RBMECs (control) were incubated on a Transwell system under normoxia. Bottom: RBMECs transfected with anti-miR-107 or scr-miR and un-transfected RBMECs (sham) were incubated on a Transwell system under OGD. The number of migrating cells was determined after 12 hours. (**H**) Quantification of the migration is expressed as the number of migrating cells per high-power field. (**I**,**J**) HUVECs. (**K**) The effect of miR-107 overexpression under normoxia on HUVECs migration was determined by scratch wound assay. Wound closure was determined after 12 hours. White lines indicate edges of scratch wounds. Representative photomicrographs showed migration. (**L**) Quantization of HUVECs migration. Data are presented as mean ± SD. *P < *0.05*, vs. control group under normoxia, ^*#*^*P* < *0.05*, vs. sham group subjected to OGD.

The Matrigel assay demonstrated that HUVECs and RBMECs transfected with miR-107-overexpressing lentiviral vector (miR-107 group) were found to have enhanced tubular formation, as indicated by increased number of branch points, and most prominently, by increased tubular length (by 7.98 fold and 4.39 fold, *P* < *0.05*), as compared with negative-control scramble lentivirus transfected cells (scr-miR group; negative control group) and un-transfected cells (control group) under normoxia (Fig. 2A–F, *P* < *0.05*). On the other hand, the miR-107-downregulating lentiviral vector (anti-miR-107) transfected HUVECs and RBMECs expressed lower levels of miR-107 resulting in reduction of tubular length by 14.67 fold and 10.11 fold under OGD (Fig. 2A–F, *P* < *0.05*). No significant effect on cell migration capacity was detected after transfection with negative-control scramble lentivirus or in un-transfected cells (Fig. 2A–F, *P* < *0.05*).

The transwell invasion assay revealed that the number of miR-107-transfected RBMECs and HUVECs penetrating the membrane was increased by 3.67 fold and 5.8 fold respectively at 12 h as compared with the scr-miR cells and un-transfected cells under normoxia (Fig. 2G–J, *P* < *0.05*). Conversely, after down-regulating the level of miR-107 in RBMECs by transfection with anti-miR-107, we found that migration of RBMECs was impeded by approximately 70% as compared with scr-miR (negative control group) and un-transfected cells (control group) under OGD at 12 h (Fig. 2G,H, *P* < *0.05*). Similarly, invasion of HUVECs was also reduced by 58.2% following anti-miR-107 transfection under OGD at 12 hours (Fig. 2I,J, *P* < *0.05*). There was no significant difference between scr-miR group and control group (Fig. 2G–J, *P* < *0.05*).

The wound healing assay showed that the migration ability of miR-107-transfected RBMECs, was significantly improved as demonstrated by increased migration distance at 12 h when compared with scr-miR transfected cells (Fig. 2K,L, *P* < *0.05*).

### Regulation of the expression of endogenousVEGF165 or VEGF164 by miR-107

**Figure 3:**
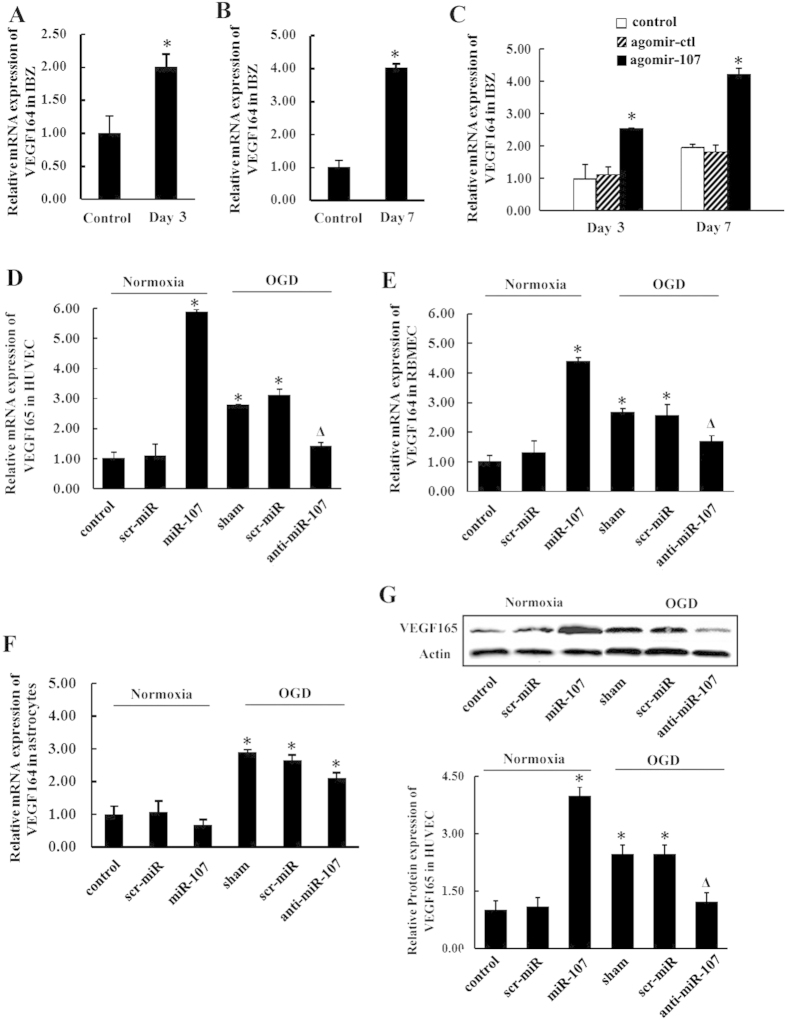
Regulation of the expression of endogenous VEGF165 or VEGF164 by miR-107. (**A**,**B**) qRT-PCR showed the mRNA levels of VEGF164 and VEGF120 in rats IBZ on 3rd and 7th days after pMCAO compared with sham-operated group (control). Data are presented as mean ± SD. *P < *0.05*, vs. control group. (**C**) Rats after pMCAO were injected with agomir (agomir-107) or negative control (scr-miR). qRT-PCR results showed that agomir-107 increased VEGF164 and VEGF120 expression in IBZ on day 3 and day 7 , compared with negative control (scr-miR) and control group. Data are presented as mean ± SD. *P < *0.05*, vs. control group (**D**) qRT-PCR data of VEGF165 in HUVECs from over-expression of miR-107 under normoxia and down-regulation of miR-107 under OGD for 12 h (**E**) qRT-PCR data of VEGF164 in RBMECs (**F**) qRT-PCR data of VEGF164 in astrocytes. (**G**) Representative pictures and analysis diagram showing the protein levels of VEGF165 in HUVECs as determined by Western blotting. Data are presented as mean ± SD. *P < *0.05*, vs. control group, ^Δ^*P* < *0.05*, vs. sham group subjected to OGD.

The mRNA expression level of VEGF164 was increased in IBZ on day 3 and day 7 after pMCAO in rats, presenting the same trend as that with miR-107 (Fig. 3A,B, *P* < *0.05*). Further qRT-PCR detection showed that up-regulation of miR-107 level by lateral ventricular injection of agomir-107, a synthetic double stranded miR-107 mimics, in pMCAO rats increased the level of VEGF164 as compared with agomir control (agomir-ctl) group on day 3 and day 7 in IBZ (Fig. 3C, *P* < *0.05*). Figure 3D,E showed that miR-107 up-regulation in HUVECs and RBMECs also raised the levels of endogenous VEGF165 or VEGF164 as compared with scr-miR. Likewise, down-regulation of miR-107 by transfection with anti-miR-107 decreased the expression of endogenous VEGF165 or VEGF164 in HUVECs and RBMECs (Fig. 3D,E, *P* < *0.05*). However, miR-107 had little effect on astrocytes in terms of VEGF164 secretion as compared with scr-miR (Fig. 3F, *P* > *0.05*). Similarly, we detected the protein expression level of VEGF165 in HUVECs. Compared with the control group and negative control group (scr-miR), the relative expression of VEGF165 (in the western blot assay) was increased in the miR-107 group and decreased in the anti-miR-107 group (Fig. 3G).

### Dicer-1 is direct target of miR-107

**Figure 4:**
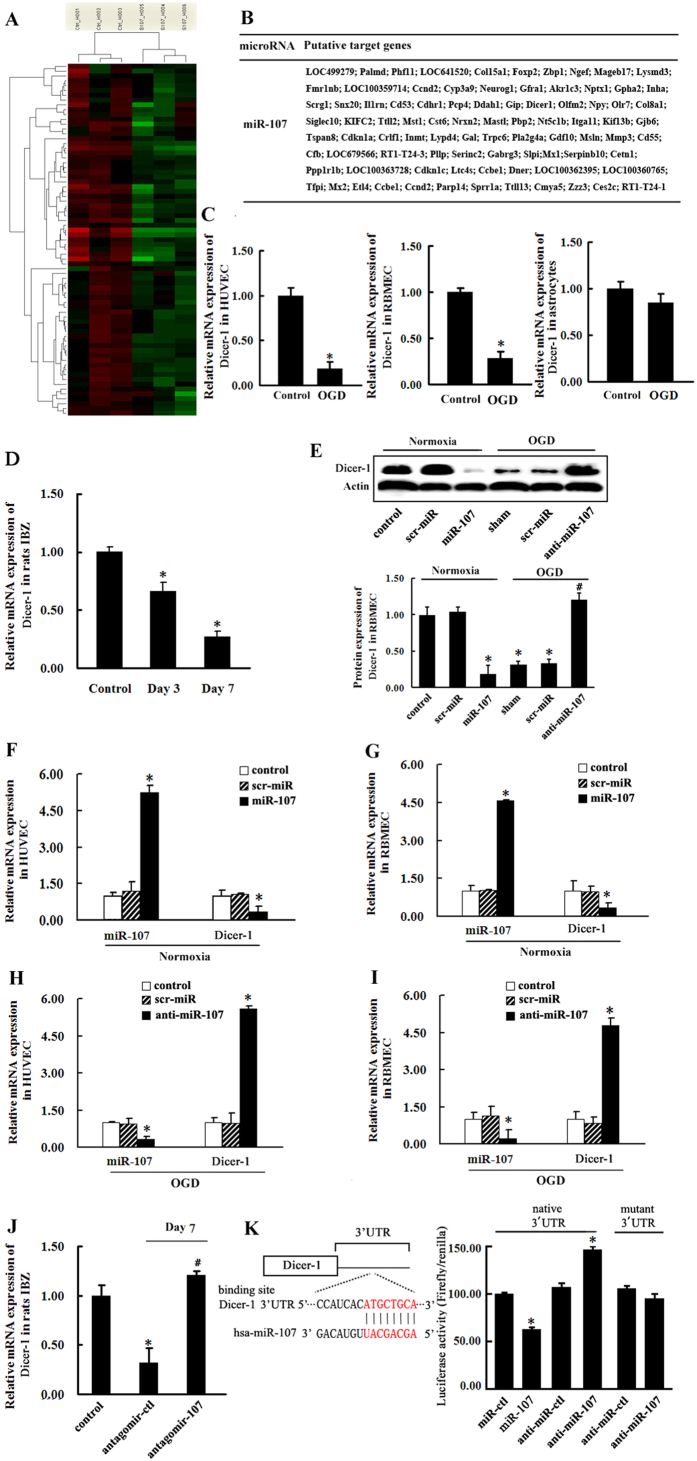
Dicer-1 is direct target of miR-107. (**A**) Heat-map of miRNAs that were differentially expressed at least 1.5-fold between RBMECs transfected with miR-107 and RBMECs transfected with scramble probe. (**B**)The putative targets genes of miR-107. (**C**) The expression of Dicer-1 in HUVECs, RBMECs and astrocytes under OGD for 12 h. *P < *0.05*, vs. control. (**D**) The mRNA levels of Dicer-1 in rat IBZ on 3rd and 7th days after pMCAO were detected by qRT-PCR. *P < *0.05*, vs. control. (**E**) Representative picture and analysis diagram show the protein levels of Dicer-1 in HUVECs as determined by Western blotting. *P < *0.05*, vs. Control, ^Δ^*P* < *0.05*, vs. sham group. (**F**,**G**) miRNA levels of miR-107 and Dicer-1 were detected in HUVECs or RBMECs transfected with miR-107 or scr-miR and in un-transfected HUVECs (control) by using qRT-PCR. (**H,I**) miRNA levels of miR-107 and Dicer-1 were detected in HUVECs or RBMECs transfected with anti-miR-107 or scr-miR and in un-transfected HUVECs (control) by using qRT-PCR. *P < *0.05*, vs. control. (**J**) After pMCAO, rats were injected with antagomir control (antagomir-ctl) or antagomir-107 and divided into 3 group: pMCAO (control), negative control (antagomir-ctl) and antagomir-107. The mRNA level of Dicer-1 in the three groups were detected by using qRT-PCR on 7th day after pMCAO. *P < *0.05*, vs. control, ^#^*P* < *0.05*, vs. antagomir-ctl group. (**K**) Luciferase activity of reporter constructs carrying luciferase cDNA and parts of the 3′UTR of target mRNAs. Left, Localization of binding sites for human miR-107 (hsa-miR-107) in the 3′UTR of target mRNA and their evolutionary conservation. Right, Quantitative analysis of 3′UTR luciferase activities. HUVECs were transfected with miR-107 or anti-miR-107 (in parallel with control molecules miR-ctl and anti-miR-ctl). Data are from 3 independent experiments performed in triplicate. Data are presented as mean ± SD. *P < *0.05*, vs. control.

To explore the molecular mechanisms involved in regulation of VEGF165/VEGF164 expression by miR-107 in endothelial cells, we examined the potential target of miR-107 using gene-chip assay (Fig. 4A), and found 96 target genes (Fig. 4B). Notably, we found that transcription factor Dicer-1 possesses a specific binding site for miR-107 by using miRanda^12^, RNAhybrid^13^ and TargetScan^14^. When subjected to OGD for 12 h, which results in upregulation of miR-107, Dicer-1 dropped in both HUVECs and RBMECs whereas there was no effect in astrocytes (Fig. 4C, *P* < *0.05*). Similarly, the mRNA level of Dicer-1 was significantly reduced in IBZ on day 3 and day 7 after pMCAO (Fig. 4D, *P* < *0.05*). To validate that Dicer-1 was the target of miR-107, HUVECs and RBMECs were transfected with miR-107. Following transfection, Dicer-1 protein expression was decreased under normoxia and its expression at mRNA level was also inhibited (Fig. 4E–I, *P* < *0.05*). On the other hand, transfection with anti-miR-107 in HUVECs and RBMECs increased Dicer-1 expression (Fig. 4E–I, *P* < *0.05*) under OGD. *In vivo*, after miR-107 expression was down-regulated by lateral ventricular injection of antagomir-107 in rats brain, Dicer-1 expression was increased on day 7 after pMCAO (Fig. 4J, *P* < *0.05*). Then, we integrated the respective 3′UTR regions of Dicer-1 into a luciferase reporter gene and determined the luciferase activity in HUVECs transfected with synthetic miR-107 precursors. We found that miR-107 significantly inhibited luciferase activity which is a measure of transcriptional activity (Fig. 4K, *P* < *0.05*). These findings indicate that Dicer-1 was a direct target of miR-107.

### miR-107 regulates the expression of endogenousVEGF165 or VEGF164 via Dicer-1

**Figure 5:**
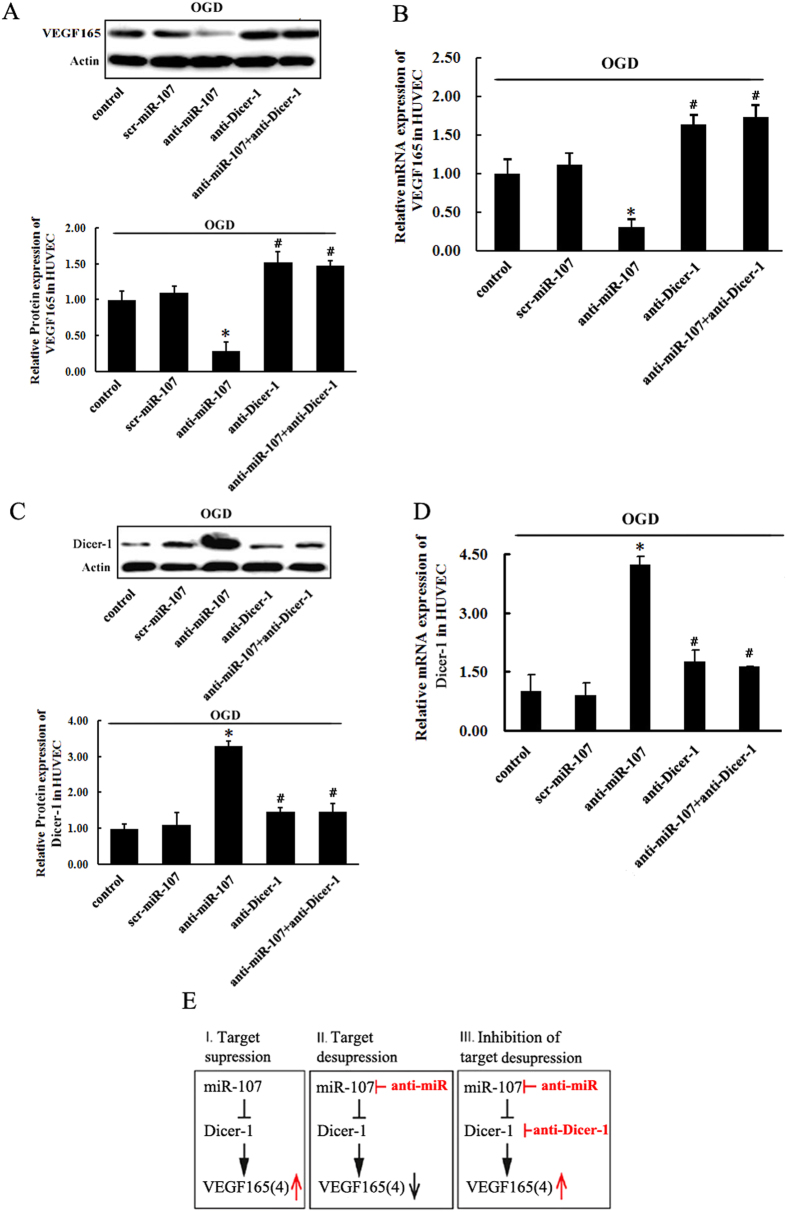
miR-107 regulates the expression of endogenous VEGF165 or VEGF164 via Dicer-1 by miR-107. (**A**) Representative picture and analysis diagram show the protein levels of VEGF165 or Dicer-1 in HUVECs as determined by Western blotting. (**B**) The expression of Dicer-1 was decrease in the miR-107-transfected HUVECs. Under hypoxia, down-regulation of Dicer-1 could strongly induce mRNA expression of VEGF165 in anti-miR-107-transfected HUVECs. (**C**,**D**) RBMECs. (**E**) Schematic illustration of target supression by miR-107 (left), target desupression by anti-miR-107 treatment (center), and inhibition of the latter by lentivirus-mediated RNA interference with Dicer-1 (right). Data are presented as mean ± SD. *P < *0.05*, vs. control group, #*P* < *0.05*, vs. anti-miR-107 group.

To confirm that miR-107 exerts its effect on angiogenesis through down-regulation of Dicer-1, we designed a strategy (Fig. 5E) to validate the functional relevance of those downstream target genes. If Dicer-1 was responsible for the effect of miR-107 on VEGF165, RNA interference with Dicer-1 expression (by lentivirus transfection) should specifically counteract the effect of anti-miR-107 in the expression of Dicer and VEGF165. This concept was tested by transfecting HUVECs *in vitro* with miR-107-downregulating lentivirus (anti-miR-107) and Dicer-1-downregulating lentivirus (anti-Dicer-1) to reduce the expression of miR-107 and Dicer-1 (Fig. 5C, *P* < *0.05*). We found that suppressed Dicer-1 expression (Fig. 5A–D, *P* < *0.05*) in anti-miR-107 transfected HUVECs under OGD resulted in strong induction of VEGF165 expression (Fig. 5A–D, *P* < *0.05*), confirming that, under hypoxia, miR-107 induced VEGF165 expression by suppressing Dicer-1.

### Therapy with miR-107 Improves Angiogenesis after pMCAO

**Figure 6:**
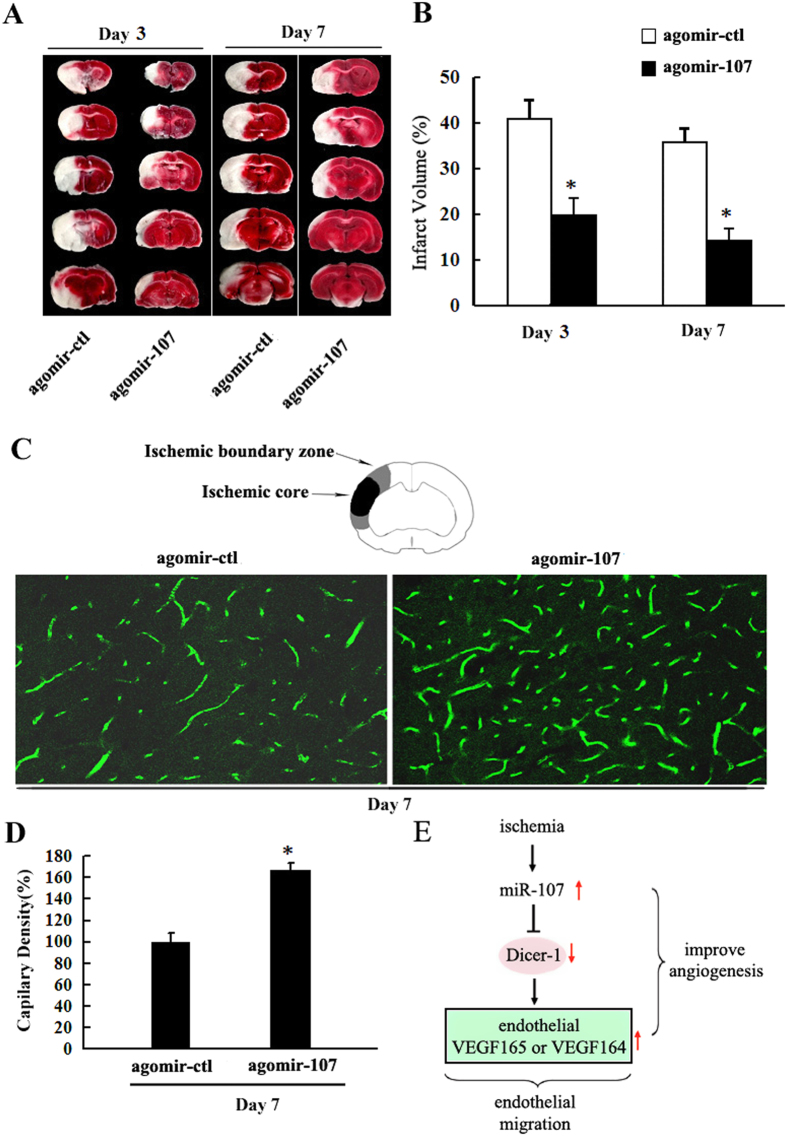
Therapy with miR-107 Improves Angiogenesis after pMCAO. (**A**) TTC staining. (**B**) Quantitative analysis showed that agomir-107 treatment significantly reduced the infarct volume as compared to the agomir negative control group (agomir-ctl). (**C**) Capillary density was evaluated by FITC tail vein injection, and then the vessel number was quantified by FITC (green). (**D**) Quantification of capillary density. Data are presented as mean ± SD. *P < *0.05*, vs. agomir-ctl group. (**E**) Scheme of miR-107-regulated target and down-stream signaling cascades.

Upregulation of miR-107 by injecting agomir-107 into lateral ventricles resulted in increased number of capillaries in IBZ as compared with agomir control group (Fig. 6A,B, *P* < *0.05*). Further detection showed that overall infarct volume in miR-107 treatment group was significantly reduced compared to agomir control group. (Fig. 6C,D, *P* < *0.05)*. A schematic summary of our findings is presented as Fig. 6E.

## Discussion

In the present study, we demonstrated that 1) miR-107 expression was significantly upregulated in the IBZ after pMCAO in rats and in ECs and astrocytes subjected to OGD for 12 h. 2) miR-107 significantly enhanced tube formation and migration of ECs resulting in increased angiogenesis both *in vitro* and *in vivo*. 3) miR-107 up-regulated the expression of endogenous VEGF165 in HUVECs (or endogenous VEGF164 in RBMECs) by directly downregulating the expression of Dicer-1. 4) miR-107 therapy significantly reduced the overall infarct volume following cerebral ischemia by improving angiogenesis.

During the post-stroke progression of angiogenesis, cells adjust their transcription and translation to adapt to the deficit in O_2_ and energy^15^. Some of these miRNAs are regulated by tissue oxygen levels and are activated by hypoxia via hypoxia-inducible factor-1 (HIF-1)^16^,^17^. A previous study demonstrated that miR-107 was a member of HRMs. Its expression is regulated by HIF1α. and had binding sites with HIF1α^5^. In the present study, we further confirmed that miR-107 was strongly induced in IBZ after pMCAO (Fig. 1A,B) and in HUVECs, RBMECs and astrocytes under OGD (Fig. 1F–H). HIF1α was also increased in HUVECs, RBMECs and astrocytes under hypoxia. The expression pattern of HIF1α was similar to that of miR-107 (supplemental Figure S1).

Previous studies mainly focused on the response of miR-107 to various stresses, but little is known about the functional consequences of elevated miR-107 expression in angiogenesis after stroke. In the present study, we demonstrated that, *in vivo,* down-regulation of miR-107 (Fig. 1E) significantly decreased the vascular density after pMCAO (Fig. 1C,D). Furthermore, miR-107 also promoted tubular formation and migration of RBMECs and HUVECs (Fig. 2G–L). Inhibition of miR-107 by subjecting HUVECs and RBMECs to OGD significantly reduced tubular length (Fig. 2A–F). These results suggested that miR-107 promoted angiogenesis both *in vivo* and *in vitro*. Recent studies have shown that VEGF165 is capable of inducing vascularization and internal vascular expansion in tumors^18^,^19^. In this study, we found that VEGF164 was increased in rat IBZ after pMCAO (Fig. 3A,B) and miR-107 promoted the expression of VEGF164 in IBZ after pMCAO (Fig. 3C).

VEGF released by endothelial cells has been reported to be crucial for vascular homeostasis^9^ and functions differently from the VEGF released by astrocytes^7^. Endothelial cell-derived VEGF promoted ECs migration and angiogenesis^8^. However, a report suggested that retinal astrocyte-derived VEGF was not essential for retinalvascularization^20^ and another study found that astrocyte-derived VEGF could damage blood-brain barrier in CNS^10^. *In vitro*, we found that mRNA expression of endogenous VEGF164 was upregulated under hypoxia in RBMECs and astrocytes as well as that of VEGF165 in HUVECs (Fig. 3D–G). Upregulation of miR-107 strongly promoted endogenous VEGF164 and VEGF165 levels in RBMECs and HUVECs respectively (Fig. 3D,E,G). However, miR-107 was found to exert limited effect on the expression of endogenous VEGF164 in astrocytes (Fig. 3F). All of these findings strongly indicated that miR-107 regulates endothelial cell-derived VEGF165 (VEGF164) levels to promote angiogenesis.

We also examined the molecular mechanism by which miR-107 regulated endothelial cell-derived VEGF165 or VEGF164 expression. 96 possible miR-107 targets were identified using gene-chip assay (Fig. 4A,B), and Dicer-1 was found to possess a specific binding site for miR-107 using miRanda^12^, RNAhybrid^13^ and TargetScan^14^. The expression of Dicer-1 was decreased in ECs under hypoxia *in vitro* (Fig. 4C) and in IBZ after pMCAO in rats (Fig. 4D). A previous research showed that expression of Dicer-1 was decreased in hypoxic pulmonary artery smooth muscle cells^21^ and in chronic hypoxia^22^. This study further confirmed the role of down-regulated Dicer-1 in angiogenesis after cerebral ischemia. Here, we showed that Dicer-1 was a direct target of miR-107 using dual luciferase reporter assay (Fig. 4K). Thus, miR-107 could impair the stability of both Dicer-1 mRNA and protein (Fig. 4E–J). Down-regulation of Dicer-1 has a protective role in the post-stroke recovery. For instance, decrease of Dicer under chronic hypoxia was an adaptive mechanism that serves to maintain concerted endothelial cellular hypoxia response through HIF1α^22^. More importantly, we found that Dicer-1 was closely related to VEGF165. When Dicer-1 was silenced, anti-miR-107 failed to decrease endogenous VEGF165 in HUVECs (Fig. 5A–D). However, in astrocytes, the expression of Dicer-1 was hardly changed under OGD as compared to normoxia (Fig. 4C). These results confirmed that miR-107 suppressed Dicer-1 expression to up-regulate endogenous (ECs derived), instead of astrocyte-derived VEGF165 (VEGF164). Moreover, as a potential protector of ischemia-induced cerebral injury, miR-107 could increase the vessel density in IBZ and thereby reduce the overall infarct volume (Fig. 6A–D).

This study demonstrated that miR-107 upregulated the expression of endogenous VEGF 165 (VEGF164) via Dicer-1 under hypoxia both *in vivo* and *in vitro*. As a result, miR-107 improved angiogenesis and reduced overall infarct volume after ischemia. Overall, our findings suggest that miR-107 may serve as a valuable therapeutic entry point for improving prognosis after stroke.

## Material and Methods

### Rat pMCAO model establishment and experimental groups division

All experimental procedures were carried out in compliance with relevant guidelines and regulations of the institutional committee of animal care and use. The protocols were also approved by the medical ethics committee of Tongji Medical College, Huazhong University of Science and Technology.

Adult male Sprague Dawley rats weighing 190–240 g were obtained from and maintained in a facility under the Animal Care and Use Committee of Tongji Medical College at Huazhong University of Science and Technology, Wuhan, China. Rats were anesthetized using 2.0 to 3.0% isoflurane and maintained using 1.0 to 1.5% isoflurane (both in 70% N_2_O/30% O_2_). During the procedures, rectal temperature was maintained at 37.3 ± 0.5 °C with a feedback-regulated heating pad. pMCAO was created as previously described^23^. In briefly, the left common carotid artery, external carotid artery and internal carotid artery of the anesthetic rats were isolated via a midline incision. After ligating the left external carotid artery with a 6-0 nylon suture, a poly-L-lysine-coated 4-0 monofilament nylon suture (Beijing Sunbio Biotech Co Ltd) was inserted from the left internal carotid artery and advanced for about 18 mm so that the origin of left MCA would be occluded. Sham-operated rats underwent identical procedures but without filament insertion. Treatment was initiated 1 h after pMCAO. To assess the effect of miR-107, single lateral ventricular injection of antagomir-107 (12 mg/kg body weight each RIBOBIO, CHINA), agomir-107 (12 mg/kg body weight each RIBOBIO, CHINA) were given respectively. Antagomir control (12 mg/kg body weight each) (negative control) was lateral cerebral ventricle injected.

### Measurement of Capillary Density

Capillaries were identified, as described previously, by intravenous injection of 0.2 ml FITC-Dextran (50 mg/ml, Sigma) 10 min before animals were sacrificed^24^. In brief, three coronal cryosections (20 μm) from each rat at bregma −0.2, −0.8, and −2.8 mm were analyzed by using a TCS SP5 multiphoton laser scanning confocal microscope (Nikon, Japan). Ten fields of view from each coronal section were collected from the IBZ after the left pMCAO. A threshold was applied to each digitized image to ensure that the number of FITC pixels reflected the original pattern of FITC-dextran-perfusion. Data were presented as a percent ratio between the number of FITC after injection of antagomir-107 or agomir-107 into rat IBZ pixels and the antagomir control.

### Cell culture

#### Primary Culture of RBMECs

RBMECs were taken from the brain tissue of SD rats (n = 3–5 weeks of age) and were primarily cultured as previously described^25^. In brief, rat brains were collected and white matter, brain stem, piamater and surface vessels were removed. The isolated cerebral cortices were minced into small pieces, homogenized in high-glucose DMEM. After 1-h digestion with 0.1% collagenase II/dispase and 500 U/ml DNase I at 37 °C, the samples were centrifuged at 500 × g for 5 min at 4 °C. The precipitate was re-suspended in 25% bovine serum album (BSA). After centrifugation at 1,000 × g for 20 min at 4 °C, the microvessels suspended in the middle layer were harvested and re-centrifuged at 500 × g for 5 min (4 °C). The microvessel pellets were then re-suspended in 8 ml of ECM (Sigma, USA), plated onto 75 cm 2 plastic flasks, and stored in an incubator in 5% humidified CO2 at 37 °C.

#### Primary Culture of Astrocytes

Astrocytes for primary culture were prepared from mice on post-natal day 1 as previously described^26^,^27^. Briefly, cerebral cortices of 1-day-old SD rats were isolated, minced and digested with trypsin (0.25 mg/ml) and DNase (0.1 mg/ml) for 20 min at 37 °C. Dissociated cells were suspended in growth medium (high-glucose DMEM) supplemented with 10% FBS and 1% penicillin/streptomycin), plated onto poly-L-lysine-coated 75 cm^2^ plastic flasks at a density of 2 × 10^5^ cells per square centimeter, and maintained at 37 °C and 5% CO_2_. Medium was exchanged every 2–3 d. After culturing for 2 weeks, microglias were detached from flasks by gentle shaking at 260 rpm. The remaining adherent astrocytes were identified morphologically under a light microscope after immuno-histochemical staining with glial fibrillary acidic protein (GFAP). Over 95% of the cells were GFAP-positive, with a density of 1 × 105 cells/cm^2^.

#### Culture of HUVECs

HUVECs were purchased from ScienCell Inc. (Carlsbad, CA, USA). The cells were grown in ECM (Sciencell, USA) supplemented with essential and non-essential amino acids, vitamins, organic and inorganic compounds, hormones, growth factors, trace minerals and a low concentration of fetal bovine serum (5%). All cells were incubated at 37 °C in 5% CO_2_.

### Short-interfering RNA (siRNA) Transfection

Cells were transfected with miR-107 overexpressing lentivirus mediated (miR-107), miR-107 down-regulating lentivirus mediated (anti-miR-107), Dicer-1-down-regulating lentivirus mediated (anti-Dicer-1) or corresponding scramble versus lentivirus (scr-miR) by using lipfectamine2000TM (Invitrogen, Carlsbad, CA, USA) according to the manufacturer’s instructions. The samples were divided into groups: miR-107 group (or anti-miR-107 group), anti-Dicer group, scr-miR group, and un-transfected group (control). Transfection efficiency was monitored by qRT-PCR. The miR-107, anti-miR-107, anti-Dicer-1 and the corresponding negative controls (scr-miR) were chemically synthesized by Genechem (Shanghai, China).

### Oxygen-Glucose Deprivation

Cells were subjected to OGD by replacing culture medium with DMEM previously saturated with 95% N_2_ and 5% CO_2_ containing 116 mM NaCl, 5.4 mM KCl, 0.8 mM MgSO_4_, 26.2 mM NaHCO_3_, 1 mM NaH2PO4, 1.8 mM CaCl_2_, and 0.01 mM glycine and cultured for 12 h in a chamber at 37 °C, in 95% N_2_ and 5% CO_2_. Control HUVECs and RBMECs were not exposed to OGD.

### qRT-PCR

Total RNA was extracted from IBZ or cells by using RNA STAT-60 kit (TEL-TEST Electronics Labs Inc., Austin, TX, and USA) according to the manufacturer’s instructions. Total RNA was reversely transcribed with a TaqMan cDNA Synthesis Kit (Applied Biosystems, Foster City, CA, USA) and amplified by using a Taqman7500 (Applied Biosystems). The data were analyzed by employing iCycler™iQ Optical System Software, Version 3.0a (Bio-Rad Laboratories, China). The primers are: VEGF165 5′-GAGGGCAGAATCATCACGAAG-3′ (forward primer) and 5′-TCC TATGTGCTGGCCTTGGTGA-3′ (reverse primer). VEGF164 5′- GCCAGCACATAGGAGAGATGAGC-3′ (forward primer) and 5′- GCGAATTCTACTACTGCTTGCTGATTCCA-3′ (reverse primer). R-Dicer-1 5′- CGATAACTTTATTGGAGATTTAC-3′ (forward primer) and 5′- GTAAATAGTGAAGGGAAATTACT-3′ (reverse primer). H-Dicer-1 5′-CGATAACTTTATTGGAGATTTAC-3′ (forward primer) and 5′-GTAAATAGTGAAGGGAAATTACT -3′ (reverse primer). R-HIF-1α 5′- CATCTCCACCTTCTACCC-3′ (forward primer) and 5′- CTCTTTCCTGCTCTGTCTG-3′ (reverse primer). H-HIF-1α 5′-TGGACATACGCAGACCCAAACC-3′ (forward primer) and 5′-GAGATACCAGCACCCAGCCAGT-3′ (reverse primer). H-β-Actin 5′-GACTACCTCATGAAGATC -3′ (forward primer) and 5′-GATCCACATCTGCTGGAA -3′(reverse primer). R-β-Actin 5′-ATGGATCCGCCAACACAGTGCTGTCTGG -3′ (forward primer) and 5′-GCGAATTCTACTACTGCTTGCTGATTCCA -3′ (reverse primer). The relative expression of mature miR-107 was calculated against U6 RNA (internal control) by using the 2^ΔΔCt^ method. The relative expression of VEGF165, VEGF121, Dicer-1 and HIF-1α was calculated against β-actin RNA (internal control) by using the 2^ΔΔCt^ method. The PCR was run along with no-template control and RT-minus control.

### Luciferase Reporter Assays

Luciferase-wt and Luciferase-mut were co-transfected with *in-vitro*-produced miR-107 or anti-miR-107 into HUVECs. Luciferase activity was measured in cell lysates 48 hours after transfection using the Dual-Light luminescent reporter gene assay kit (Applied Biosystems). Results were normalized against β-galactosidase activity.

### Western blot analysis

Equal amount of protein samples (30 μg) extracted from HUVECs were separated on a 10%–15% SDS/polyacrylamide gel (SDS/PAGE), and transferred onto PVDF membranes. After blotting, membranes were blocked in TBS-Tween buffer containing 20 mM Tris-HCl, 5% nonfat milk, 150 mM NaCl, and 0.05% Tween-20 (pH 7.5) for 1 h at 21 °C, then incubated with primary antibodies against Dicer-1 (abcom, 1:1000), VEGF165 (RD, 1:500)and β-actin (abcom, 1:1000) overnight at 4 °C, and finally incubated with secondary antibody (abcom, 1:3000). Actin served as protein loading control. The expression of Dicer-1 and VEGF165 protein was determined by using Image J software. Three independent experiments were carried out in order and the mean was used as final result.

### Observation of Tube Formation

Basement membrane matrix (Matrigel, BD Biosciences) was thawed at 4 °C, and 300 μl was added into each well of a 24-well plate. The Matrigel-coated plate was incubated for 30 min at 37 °C to polymerize the Matrigel. BMECs and HUVECs transfected with miR-107, anti-miR-107 or scr-miR (10^4^ cells/100 μl) and un-transfected RBMECs and HUVECs (10^4^ cells/100 μl) suspended in ECM were plated into a coated well, and incubated for 24 h at 37 °C. Tube length was quantitatively measured on pictures captured (200×). Each sample was examined in three randomly selected fields, and the examination was repeated three times.

### Wound Healing Assay

HUVECs transfected with miR-107, anti-miR-107 or scr-miR and un-transfected HUVECs (5 × 10^5^ per well) were seeded into 24-well plates and allowed to adhere for 24 h. Artificial wounds were made by cutting the monolayer cells with a 200-μl pipette tip. Afterwards, 1 × PBS was used to remove cell debris and floating cells. Fresh serum-free medium was added, and the cells were allowed to heal for 24 h. Photographs were taken at the same site, 0 and 24 h after the injury. The healing of the wounds was assessed by measuring the wound gap.

### Transwell invasion assay

Cell invasion assays were performed in 24-well transwells (8 mm pore size, Corning Life Sciences) coated with matrigel (1 mg/ml, BD Sciences) as previously described^28^. RBMECs and HUVECs transfected with miR-107, anti-miR-107 or scr-miR (10^4^/well) and un-transfected RBMECs and HUVECs (10^4^/well) were seeded into the upper chambers of the wells in 200 μl DMEM, and the lower chambers were filled with 500 μl ECM medium, used for inducing cell migration. After incubation for 24 h, the cells on the filter surface were fixed with 4% formaldehyde, stained with 0.5% crystal violet, and examined under a microscope. Cells on at least 6 randomly chosen microscopic fields (200×) were counted.

### Measurement of infarct volume and neurological deficit

Infarct volume was measured by using 2% 2, 3, 5-triphenyltetrazolium chloride (TTC). Rat brains were removed 3 and 7 days after pMCAO, and sliced into coronal sections (1 mm in thickness). The slices were stained with 2% TTC for 15 min at 37 °C, scanned and the infarct area was estimated by using the Metamorph software package. The infarct volume was calculated using a derived formula^29^.

## Additional Information

**How to cite this article**: Li, Y. *et al.* MicroRNA-107 contributes to post-stroke angiogenesis by targeting Dicer-1. *Sci. Rep.* **5**, 13316; doi: 10.1038/srep13316 (2015).

## Supplementary Material

Supplementary Information

## References

[b1] Ergul A., Alhusban A. & Fagan S. C. Angiogenesis: a harmonized target for recovery after stroke. Stroke. 43, 2270–2274 (2012). 22618382 10.1161/STROKEAHA.111.642710 PMC3404267

[b2] Yin K.-J. *et al.* Vascular endothelial cell-specific microRNA-15a inhibits angiogenesis in hindlimb ischemia. J Biol Chem. 287, 27055–27064 (2012). 22692216 10.1074/jbc.M112.364414 PMC3411046

[b3] Muramatsu F., Kidoya H., Naito H., Sakimoto S. & Takakura N. microRNA-125b inhibits tube formation of blood vessels through translational suppression of VE-cadherin. Oncogene. 32, 414–421 (2012). 22391569 10.1038/onc.2012.68

[b4] Chen P.-S. *et al.* miR-107 promotes tumor progression by targeting the let-7 microRNA in mice and humans. J Clin Invest. 121, 3442 (2011). 21841313 10.1172/JCI45390 PMC3163949

[b5] Chen Z. *et al.* Hypoxia-responsive miRNAs target argonaute 1 to promote angiogenesis. J Clin Invest. 123, 1057 (2013). 23426184 10.1172/JCI65344 PMC3582133

[b6] Otsuka M. *et al.* Impaired microRNA processing causes corpus luteum insufficiency and infertility in mice. J Clin Invest. 118, 1944 (2008). 18398510 10.1172/JCI33680 PMC2289794

[b7] Carmeliet P. & Jain R. K. Molecular mechanisms and clinical applications of angiogenesis. Nature. 473, 298–307 (2011). 21593862 10.1038/nature10144 PMC4049445

[b8] Guangqi E. *et al.* Endogenous vascular endothelial growth factor-A (VEGF-A) maintains endothelial cell homeostasis by regulating VEGF receptor-2 transcription. J Biol Chem. 287, 3029–3041 (2012). 22167188 10.1074/jbc.M111.293985 PMC3270960

[b9] Lee S. *et al.* Autocrine VEGF signaling is required for vascular homeostasis. Cell. 130, 691–703 (2007). 17719546 10.1016/j.cell.2007.06.054 PMC3010851

[b10] Argaw A. T. *et al.* Astrocyte-derived VEGF-A drives blood-brain barrier disruption in CNS inflammatory disease. J Clin Invest. 122, 2454 (2012). 22653056 10.1172/JCI60842 PMC3386814

[b11] Weidemann A. *et al.* Astrocyte hypoxic response is essential for pathological but not developmental angiogenesis of the retina. Glia. 58, 1177–1185 (2010). 20544853 10.1002/glia.20997 PMC2993327

[b12] John B. *et al.* Human microRNA targets. PLoS Biol. 2, e363 (2004). 15502875 10.1371/journal.pbio.0020363 PMC521178

[b13] Krek A. *et al.* Combinatorial microRNA target predictions. Nat Genet. 37, 495–500 (2005). 15806104 10.1038/ng1536

[b14] Michon F. Tooth evolution and dental defects: From genetic regulation network to micro‐RNA fine‐tuning. Birth Defects Res Part A: Clin Mol Teratol. 91, 763–769 (2011). 21591243 10.1002/bdra.20787

[b15] Fraisl P., Mazzone M., Schmidt T. & Carmeliet P. Regulation of angiogenesis by oxygen and metabolism. Dev Cell. 16, 167–179 (2009). 19217420 10.1016/j.devcel.2009.01.003

[b16] Wang F. *et al.* miR-210 directly suppresses BNIP3 expression to protect against the hypoxia-induced apoptosis of neural progenitor cells. Stem cell research. 11, 657–67 (2013). 23688833 10.1016/j.scr.2013.04.005

[b17] Czyzyk-Krzeska M. F. & Zhang X. MiR-155 at the heart of oncogenic pathways. Oncogene. 33, 677–8 (2013). 23416982 10.1038/onc.2013.26 PMC4047641

[b18] Reddy C. L., Yosef N. & Ubogu E. E. VEGF-A165 Potently induces human blood–nerve barrier endothelial cell proliferation, angiogenesis, and wound healing *in vitro*. Cell Mol Neurobiol. 33, 789–801 (2013). 23712256 10.1007/s10571-013-9946-3 PMC3723762

[b19] Catena R. *et al.* Increased expression of VEGF121/VEGF165–189 ratio results in a significant enhancement of human prostate tumor angiogenesis. Int J Cancer. 120, 2096–2109 (2007). 17278099 10.1002/ijc.22461

[b20] Scott A. *et al.* Astrocyte-derived vascular endothelial growth factor stabilizes vessels in the developing retinal vasculature. PLoS One. 5, e11863 (2010). 20686684 10.1371/journal.pone.0011863 PMC2912336

[b21] Wu C. *et al.* Hypoxia potentiates microRNA-mediated gene silencing through posttranslational modification of Argonaute2. Mol. Cell. Biol. 31, 4760–4774, 10.1128/mcb.05776-11 (2011). 21969601 PMC3232924

[b22] Ho J. J. *et al.* Functional importance of Dicer protein in the adaptive cellular response to hypoxia. J. Biol. Chem. 287, 29003–29020 (2012). 22745131 10.1074/jbc.M112.373365 PMC3436557

[b23] He Y. *et al.* Effects of cerebral ischemia on neuronal hemoglobin. J Cereb Blood Flow Metab. 29, 596–605 (2008). 19066615 10.1038/jcbfm.2008.145 PMC2683405

[b24] Zhang L. *et al.* Adjuvant treatment with a glycoprotein IIb/IIIa receptor inhibitor increases the therapeutic window for low-dose tissue plasminogen activator administration in a rat model of embolic stroke. Circulation. 107, 2837–2843 (2003). 12756151 10.1161/01.CIR.0000068374.57764.EB

[b25] Kim J. A., Tran N. D., Wang S.-J. & Fisher M. J. Astrocyte regulation of human brain capillary endothelial fibrinolysis. Thromb Res. 112, 159–165 (2003). 14967413 10.1016/j.thromres.2003.10.021

[b26] Xia Y. P. *et al.* The protective effect of sonic hedgehog is mediated by the propidium iodide 3-kinase/AKT/Bcl-2 pathway in cultured rat astrocytes under oxidative stress. Neuroscience. 209, 1–11 (2012). 22402346 10.1016/j.neuroscience.2012.02.019

[b27] Li Y. *et al.* Sonic hedgehog (Shh) regulates the expression of angiogenic growth factors in oxygen-glucose-deprived astrocytes by mediating the nuclear receptor NR2F2. Mol. Neurobiol. 47, 967–975 (2013). 23378030 10.1007/s12035-013-8395-9

[b28] Zhang C. *et al.* Inhibitory effects of microRNA-34a on cell migration and invasion of invasive urothelial bladder carcinoma by targeting notch1. Journal of Huazhong University of Science and Technology. 32, 375–382 (2012). 22684561 10.1007/s11596-012-0065-z

[b29] Swanson R. A. *et al.* A semiautomated method for measuring brain infarct volume. J Cereb Blood Flow Metab. 10, 290–293 (1990). 1689322 10.1038/jcbfm.1990.47

