# Supplementary material for: MicroRNA-107 contributes to post-stroke angiogenesis by targeting Dicer-1
Source: Sci Rep. 2015 Aug 21;5:13316. doi: 10.1038/srep13316 (PMC4543985; doi:10.1038/srep13316)
Supplement: Supplementary Information [file srep13316-s1.pdf]

SUPPLEMENTAL MATERIAL

MicroRNA-107 Contributes to Post-stroke Angiogenesis by Targeting Dicer-1

Short title：miR-107 regulates angiogenesis via Dicer-1

YananLi[1]*,Ling Mao[1]*, Yuan Gao[1], Suraj Baral[1], Yifan Zhou[1], &Bo Hu[1 #]

[1]Department of Neurology, Union Hospital, Tongji MedicalCollege, Huazhong University of Science and Technology,Wuhan 430022, China

# Correspondence to Bo Hu, Department of Neurology, Union Hospital, Tongji Medical College, Huazhong University of Science and Technology, Wuhan 430022, China.

Tel:+86-13707114863; Fax: +86-27-85726028;

E-mail: hubo@mail.hust.edu.cn

The first two authors(*)contributed equally to this work.

Total words: 5632

Cell culture

Primary Culture of Rat Brain Microvascular Endothelial Cells

Rat brain microvascular endothelial cells were taken from the brain tissue of SD rats (n=3-5 weeks of age) and subjected to primarily cultured, as previously described.[1] In brief, rat brains were collected and removed of white matter, brain stem, piamater and surface vessels. The isolated cerebral cortices were minced into small pieces, homogenized in high-glucose DMEM. After 1-h digestion with 0.1 % collagenase II/dispase and 500U/ml DNase I at 37 ℃, the samples were centrifuged at 500 ×g for 5 min at 4 ℃. The precipitate was re-suspended in 25 % bovine serum album (BSA). After centrifugation at 1,000 ×g for 20 min at 4 ℃, the microvessels suspended in the middle layer were harvested and re-centrifuged at 500 ×g for 5 min (4 ℃). The microvessel pellets were then re-suspended in 8 ml of ECM (Sigma, USA), plated onto 75 cm2 plastic flasks, and stored in an incubator in 5 % humidified CO2 at 37 ℃.

Primary Culture of Astrocytes

Astrocytes for primary culture were prepared from mice at post-natal day 1 as previously described.[2] Briefly, cerebral cortices of 1-day-old SD rats were isolated , minced and digested with trypsin (0.25 mg/ml) and DNase (0.1 mg/ml) for 20 min at 37 ℃. Dissociated cells were suspended in growth medium (high-glucose DMEM) supplemented with 10 % FBS and 1 %

penicillin/streptomycin), plated onto poly-L-lysine-coated 75 cm^2 plastic flasks at a density of 2×10^5 cells per square centimeter, and maintained at 37℃ and 5% CO_2. Medium was exchanged every 2–3 d. After culturing for 2 weeks, microglias were detached from flasks by gentle shaking at 260 rpm. The remaining adherent astrocytes were identified morphologically under a light microscope after immunohistochemical staining with glial fibrillary acidic protein (GFAP). Over 95% of the cells were GFAP-positive, with a density of 1×105 cells /cm^2.

Culture of HUVECs

Human umbilical vein endothelial cells (HUVECs) were purchased from ScienCell Inc. (Carlsbad, CA, USA). The cells were grown in ECM (Sciencell, USA) supplemented with essential and non-essential amino acids, vitamins, organic and inorganic compounds, hormones, growth factors, trace minerals and a low concentration of fetal bovine serum (5%). All cells were incubated at 37 ℃ in 5% CO_2.

Oxygen-Glucose Deprivation

Cells were subjected to OGD by replacing culture medium with DMEM previously saturated with 95 % N_2 and 5% CO_2 containing 116 mMNaCl, 5.4 mMKCl, 0.8 mM $MgSO_4$, 26.2 mM $NaHCO_3$, 1 mM NaH2PO4, 1.8 mM $CaCl_2$, and 0.01 mM glycine and cultured for 12 h in a

chamber at 37 ℃, in 95% N_2 and 5% CO_2. Control HUVECs and RBMECs were not exposed to OGD.

Quantitative real-time PCR (qRT-PCR)

Total RNA was extracted from IBZ or cells by using RNA STAT-60 kit (TEL-TEST Electronics Labs Inc., Austin, T X, and USA) according to the manufacturer's instructions. Total RNA was reversely transcribed with a TaqMancDNASynthesisKit (Applied Biosystems, Foster City, CA, USA) and amplified by using a Taqman7500 (Applied Biosystems). The data were analyzed by employing iCycler™iQ Optical System Software, Version 3.0a (Bio-Rad Laboratories, China).The primers are:R-HIF-1α 5′-CATCTCCACCTTCTACCC-3′(forward primer) and 5′-CTCTTTCCTGCTCTGTCTG-3′(reverse primer). H-HIF-1α 5′-TGGACATACGCAGACCCAAACC-3′(forward primer) and 5′-GAGATACCAGCACCCAGCCAGT-3′(reverse primer).H-β-Actin 5′-GACTACCTCATGAAGATC -3′(forward primer) and 5′-GATCCACATCTGCTGGAA -3′(reverse primer).R-β-Actin 5′-ATGGATCCGCCAACACAGTGCTGTCTGG -3′(forward primer) and 5′-GCGAATTCTACTACTGCTTGCTGATTCCA -3′(reverse primer).The relative expression of HIF-1αwas calculated against β-actin RNA (internal control) by using the $2^{\Delta\Delta Ct}$ method. The PCR was run along with no-template control and RT-minus control.

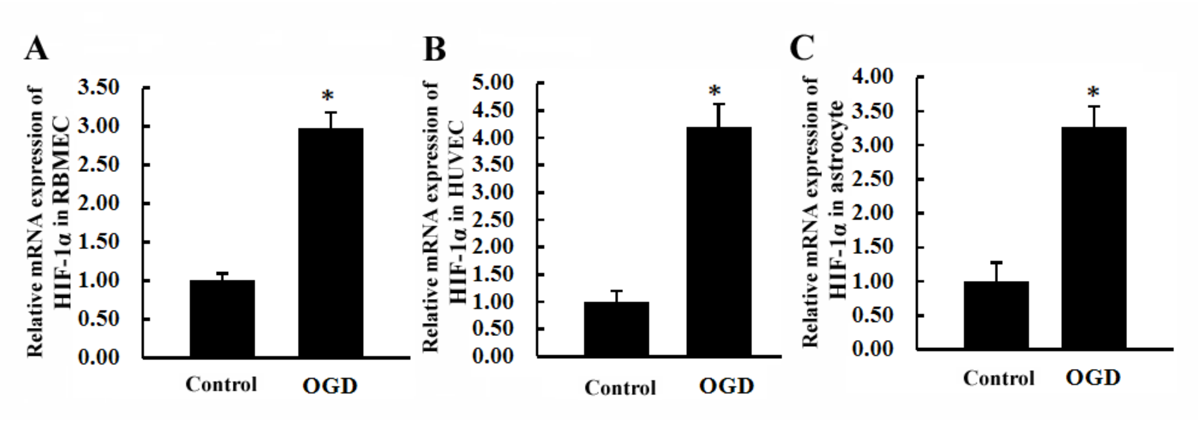

Suppl. Figure I

Expression of HIF1α. (A) Exposure to OGD for 12 h increases HIF1α expression in RBMECs by using RT-PCR. (B) HUVECs. (C) Astrocytes. Data are presented as mean ± SD. *$p < 0.05$, vs. Control group.

1 Kim, J. A., Tran, N. D., Wang, S.-J. & Fisher, M. J. Astrocyte regulation of human brain capillary endothelial fibrinolysis. *Thromb Res* **112**, 159-165 (2003).

2 Xia, Y. P. *et al*. The protective effect of sonic hedgehog is mediated by the propidium iodide 3-kinase/AKT/Bcl-2 pathway in cultured rat astrocytes under oxidative stress. *Neuroscience* **209**, 1-11 (2012).
